# Intermedin in rat uterus: changes in gene expression and peptide levels across the estrous cycle and its effects on uterine contraction

**DOI:** 10.1186/1477-7827-11-13

**Published:** 2013-02-25

**Authors:** Chi-Wai Wong, Wai-Sum O, Fai Tang

**Affiliations:** 1 Departments of Physiology, Li Ka Shing Faculty of Medicine, The University of Hong Kong, Hong Kong SAR, Pokfulam, China; 2 Department of Anatomy, Li Ka Shing Faculty of Medicine, The University of Hong Kong, Hong Kong SAR, Pokfulam, China; 3 Center of Growth, Reproduction and Development, Li Ka Shing Faculty of Medicine, The University of Hong Kong, Hong Kong SAR, Pokfulam, China; 4 Center of Heart, Brain, Hormone and Healthy Aging, Li Ka Shing Faculty of Medicine, The University of Hong Kong, Hong Kong SAR, Pokfulam, China

**Keywords:** Intermedin, Estrous cycle, Uterine contraction

## Abstract

**Background:**

The present study demonstrates the expression of intermedin (IMD) and its receptor components in the uterus of the female rat during the estrous cycle and its effect on uterine contraction.

**Methods:**

The gene expression level of intermedin and its receptor components and the peptide level of intermedin were studied by real-time RT-PCR and enzyme immunoassay (EIA) respectively. The separation of precursor and mature IMD was studied by gel filtration chromatography and EIA. The localization of IMD in the uterus was investigated by immunohistochemistry. The effect of IMD on *in vitro* uterine contraction was studied by organ bath technique.

**Results:**

Uterine mRNAs of *Imd* and its receptor components and IMD levels displayed cyclic changes across the estrous cycle. *Imd* mRNA level was the highest at proestrus while the IMD level was the highest at diestrus. IMD was found in the luminal and glandular epithelia and IMD treatment significantly reduced the amplitude and frequency of uterine contraction but not the basal tone. Both calcitonin gene-related peptide (CGRP) receptor antagonist hCGRP8-37 and adrenomedullin (ADM) receptor antagonist hADM22-52 partially abolished the inhibitory effect of IMD on uterine contraction while the specific IMD receptor antagonist hIMD17-47 completely blocked the actions. The enzyme inhibitors of NO (L-NAME) and PI3K (Wortmannin) pathways diminished the IMD effects on uterine contraction while the cAMP/PKA blocker, KT5720, had no effect, indicating an involvement of NO and PI3K/Akt but not PKA.

**Conclusions:**

IMD and the gene expression of its receptor components are differentially regulated in the uterus during the estrous cycle and IMD inhibits uterine contraction by decreasing the amplitude and frequency.

## Background

Intermedin (IMD), calcitonin, calcitonin gene-related peptide (CGRP), adrenomedullin (ADM), and amylin, all belong to the calcitonin/calcitonin gene-related peptide family [1-7]. IMD (also known as adrenomedullin 2 or ADM2), evolved early in vertebrates, is a 47-amino-acid peptide sharing about 28% and <20% homology with ADM and CGRP respectively in the middle region of the peptides [2,3]. Similar to ADM, IMD signals through the calcitonin receptor-like receptor (CRLR)/receptor activity-modifying proteins (RAMPs) receptor complexes [1-3]. However, ADM shows a preferential stimulation of receptors formed by the coexpression of CRLR with RAMP2 or RAMP3, whereas IMD is a nonselective agonist for all these receptors [1-3].

Produced from the prointermedin molecule are three molecular species of IMD which are biologically active i.e. IMD_1-53_, IMD_1-47_ and IMD_8-47_[7]. IMD has several functions similar to those of ADM. Both IMD and ADM are potent vasodilators [2,8,9] and are anorexogenic [2,4,10]. Both suppress stomach emptying [2,11,12] and increase circulating prolactin levels [2,13,14]. However, IMD also has its own effects not shared by ADM [15-21]. The effects of IMD on the cardiovascular system have been extensively investigated [7,16-18,21] whereas the study of IMD in reproduction has been confined to oocyte regulation [22], trophoblast invasion and migration [15,19], and embryonic development [20].

Both uterine ADM expression [23,24] and the response of uterine contraction to ADM [25,26] have been investigated. Uterine endometrium has been shown to have more ADM than the myometrium in rats [23], and in humans [27]. Rat uterine endometrial ADM expression peaks at proestrus and estrus and is regulated by estrogen [23,24].

Our laboratory has previously investigated the expression and functions of ADM in the reproductive system [28-38]. In the female rat, the gene expression and peptide levels of ADM change during the estrous cycle [33]. ADM inhibits ovarian steroidogenesis [33], stimulates ciliary beating but inhibits muscle contraction in the oviduct [35], and plays important roles in pregnancy [34,36]. As ADM is known to inhibit uterine contraction [25,26], we have investigated the changes of IMD gene expression and peptide levels in the uterus during the estrous cycle and its effect on spontaneous uterine contraction to understand the possible roles of IMD in the uterus.

## Methods

### Animals

Female Sprague–Dawley (SD) rats (12–13 wk) were obtained from the Laboratory Animal Unit, LKS Faculty of Medicine, the University of Hong Kong. The rats were housed at a constant temperature and humidity, under a 12-h light–dark cycle (dark period 07:00h to 19:00h), with water and rat chow *ad libitum*. Vaginal smears were obtained daily from 36 rats, and 28 rats that showed a regular 4-day estrous cycle were included in this study. Staging of the cycle into estrus and diestrus was based on the cytology of the vaginal smear. All rats were killed between 10:00 a.m. and 12:00 noon and the uteri were collected and snap-frozen in liquid nitrogen for storage at −80°C until further analysis. Immature rats injected with gonadotropin were used for the contraction study (please see a later section). All procedures had been approved by the Committee on the Use of Live Animals for Teaching and Research, the University of Hong Kong.

### RT-PCR of *Imd*, *Crlr*, and *Ramps*

Total RNA of the uterus was obtained by homogenization in TRIZOL reagent (Life Technologies, Carlsbad, CA, USA) using a polytron (Kinematica, Switzerland) [34] and subjected to RT-PCR. RNA samples (5 μg) were reverse transcribed into complementary DNA (cDNA) with the SuperScript II reverse transcriptase (Life Technologies, Carlsbad, CA). The real time RT-PCR technique has been previously described [35]. Polymerase chain reactions (PCR) were conducted by an iCycler iQ real-time PCR detection system (Bio-Rad Laboratories, Hercules, CA, USA) using iQ SYBR Green Supermix (Bio-Rad Laboratories, Hercule, CA). Three house-keeping genes (ribosomal protein L19, β-actin, and 18S ribosomal RNA) were tested and β-actin (*Actb*) was used as an internal standard based on its uniform expression across the groups. Standard curves for each primer pair were prepared by the serial dilution of cDNA to determine the PCR efficiency. The PCR efficiencies for *Imd*, *Crlr*, *Ramp1*, *Ramp2*, *Ramp3* and *Actb* (β-actin used as an internal standard) were all above 0.95. The relative gene expression levels were then analyzed by the ΔΔCt method [39], where Ct is the cycle threshold. The reaction mixtures contained 10 μl iQ SYBR Green Supermix (Bio-Rad Laboratories, Hercules, CA, USA), 2 μl template cDNA, 100 nM of each primer, and DNase-free water (Life Technologies, Carlsbad, CA, USA) to a final volume of 20 μl. Cycle conditions were 95°C for 5 min, followed by a maximum of 40 cycles of 95°C for 15 sec, 59°C for 15 sec, and 72°C for 15 sec, and extension at 72°C for 10 minutes. The reaction was completed with a dissociation step for melting point analysis with 50°C to 95°C (in increments of 0.5°C) for 10 sec each. The design of the primers was based on the published sequences (The details of the primers used and the sizes of the amplicons are shown in Table 1). Melt curve analysis for each primer showed only one peak for each product. The identities of all the PCR products were confirmed by gene sequencing (Tech Dragon Limited, Hong Kong).

**Table 1:** Gene primer sequences and GenBank accession number of rat for the real-time PCR

**Genes**	** Primer Sequences**	**GenBank Accession Number**	**Product Size (bp)**
*Imd*	**F: 5**^′^**-**GCTGATGGTCACGGTAAC**-3**^′^	**NM_201426.1**	**122**
	**R: 5**^′^**-**CGCTGGAAGGAATCTTGG**-3**^′^		
*Crlr*	**F: 5**^′^**-**CCAAACAGACTTGGGAGTCACTAGG**-3**^′^	**NM_012717.1**	**323**
	**R: 5**^′^**-** GCTGTCTTCTCTTTCTCATGCGTGC**-3**^′^		
*Ramp1*	**F: 5**^′^**-** CACTCACTGCACCAAACTCGTG**-3**^′^	**NM_031645.1**	**196**
	**R: 5**^′^**-** CAGTCATGAGCAGTGTGACCGTAA**-3**^′^		
*Ramp2*	**F: 5**^′^**-** AGGTATTACAGCAACCTGCGGT**-3**^′^	**NM_031646.1**	**163**
	**R: 5**^′^**-** ACATCCTCTGGGGGATCGGAGA**-3**^′^		
*Ramp3*	**F: 5**^′^**-** ACCTGTCGGAGTTCATCGTG**-3**^′^	**NM_020100.2**	**180**
	**R: 5**^′^**-** ACTTCATCCGGGGGGTCTTC**-3**^′^		
*Actb*	**F: 5**^′^**-** GGAAATCGTGCGTGACATTA**-3**^′^	**NM_031144.2**	**183**
	**R: 5**^′^**-** AGGAAGGAAGGCTGGAAGAG**-3**^′^		

### Measurement of IMD in the uterus

Each tissue sample (0.03 g) was homogenized in 3 ml 2N acetic acid (1 ml/0.01 g tissue, BDH Laboratory Supplies, Poole, England) and then boiled for 10 min. A 50-μl aliquot was taken for the protein assay and the remaining homogenate was centrifuged at 18600 X g for 20 min at 4°C (Sorvall SM 24; Thermo Fisher Scientific, Inc., Waltham, MA). The supernatants were all lyophilized and stored at −20°C until assay.

The lyophilized tissue samples were reconstituted in 1X IMD assay buffer. IMD level was measured with an IMD (1–50) (human) EIA kit (Phoenix Pharmaceuticals, Inc., Burlingame, CA, USA). The minimum detectable concentration was 0.26 ng/ml and the range was 0-100 ng/ml. The intra-assay and inter-assay coefficients of variation were <10% and <15% respectively. The amount of protein in each sample was measured with a protein assay reagent (BioRad, Hercules, CA, USA) spectrophotometrically at 595 nm (LKB Ultraspec II; Biochem, Berlin, Germany). The immunoreactive IMD was expressed as pg/mg protein.

### Gel filtration chromatography of the uterus

The tissues were extracted with a polytron in 1N acetic acid (BHD Laboratory Supplies, Poole, England) on ice (see above). A 50-μl aliquot of the homogenate was stored at −20°C until protein assay. The lyophilized tissue samples were reconstituted in Milli-Q water and centrifuged at 13000 rpm for 20 min at 4°C. Glacial acetic acid (96%) (Sigma, St. Louis, MO, USA) was added to the supernatant to a final concentration of 1 N acetic acid. The samples (in 500 μl of 1 N acetic acid) were then loaded on a Bio-gel P30 (Bio-Rad, Hercules, CA, USA) column (0.9 X 60 cm) and the column was eluted with 1N acetic acid at a flow rate of 1 ml/10 min for a total of 400 min. One-millilitre fractions were lyophilized and measured for IMD immunoreactivities as stated before. The level of immunoreactive IMD was expressed in terms of pg/ml of fraction/mg protein. Authentic IMD_1-53_, IMD_1-47_ and IMD_8-47_ (1 ng each, Phoenix Pharmaceuticals, Inc., Burlingame, CA, USA) were loaded on the same column as markers.

### Immunohistochemistry

To localize IMD in the uterus, a Vectastain ABC kit (Vector Laboratories, Burlingame, CA, USA) was used for the avidin-biotin histochemical staining procedure. The uterine tissues were fixed in neutral buffered formalin overnight. Paraffin-embedded sections of 5 μM thickness were dewaxed, rehydrated and then treated with 3% hydrogen peroxide in phosphate buffer saline for 30 min, followed by overnight incubation with 1:1000 diluted primary antibody of IMD (Phoenix Pharmaceuticals, Inc., Burlingame, CA, USA) at 4°C. After washing, 1:200 biotinylated secondary antibody was added, followed by preformed ABC reagent (Rabbit ABC Staining System, Santa Cruz, U.S.A.). Diaminobenzidine was used to visualize the avidin-biotin-peroxidase complex for 5 to 10 min.

### *In vitro* contraction experiment by an organ-bath technique

Immature female SD rats (21–23 days) were treated with 30 IU pregnant mare’s serum gonadotropin (PMSG) 48 h prior to the collection of tissues to simulate the estrus stage (when the spontaneous contraction is the greatest). Uteri from the rats were isolated and rinsed in Kreb’s solution (115mM NaCl, 4.7mM KCl, 1mM MgSO_4_, 15mM NaHCO_3_, 1.2mM NaH_2_PO_4_, 10.5mM glucose, and 1.6mM CaCl_2_) immediately [25,26,35]. The entire uterus [25] was then tied, via silk threads, to a tissue holder in a 10-ml organ bath containing Kreb’s solution aerated with a mixture of oxygen and carbon dioxide (95:5%) at a constant temperature of 37°C. The tissue holder was attached to a force transducer coupled to a graph recorder. As a pilot study using 1, 10 and 100 nM IMD indicated that the uterine preparation only responded to 100 nM IMD, the response to 100 nM IMD (human IMD_1-53_) was studied after 45-min equilibration. For the study on receptor antagonism, the uteri were preincubated with 1 μM hADM_22-52_ (ADM receptor antagonist), hCGRP_8-37_ (CGRP receptor antagonist), or hIMD_17-47_ (IMD receptor antagonist) or the vehicle for 1h, before the addition of 100 nM IMD. For the signaling pathways, the uteri were preincubated with KT5720 (1 μM, protein kinase A (PKA) inhibitor), N-nitro-L-arginine methyl ester (L-NAME) (100 μM, nitric oxide (NO) synthase inhibitor), or Wortmannin (1 μM, serine-threonine kinase/phosphoinositide 3-kinase (Akt/PI3K) inhibitor) (all from Sigma Chemicals, St. Louise, MO, USA.) before IMD (100 nM) was added.

### Statistical analysis

All the data were expressed as mean ± standard error of the mean (SEM), and statistical significance was assessed by one-way analysis of variance (ANOVA) followed by Student-Newman-Keuls (SNK) test for post hoc comparisons, with P<0.05 taken as significant.

## Results

### IMD immunoreactivity and mRNA level of *Imd*

**Figure 1:**
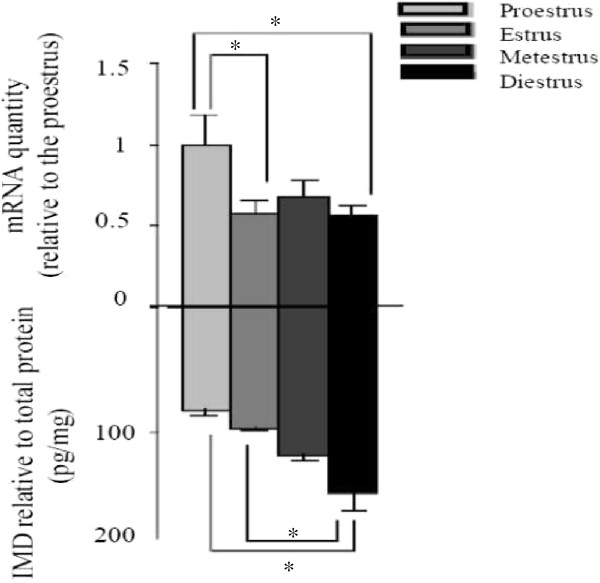
***Imd *mRNA and IMD levels in the uterus across estrous cycle. **n=6, 6, 7, and 9 for the four stages respectively. The mRNA levels were normalized to *Actb* mRNAs, and each value represents the mean ± SEM. * P<0.01.

The levels of IMD and *Imd* mRNA in the uterus of cycling rats were estimated by IMD EIA and real-time RT-PCR respectively, and the results are shown in Figure 1. The *Imd* mRNA level at the proestrus stage was taken as 1 and it was higher than those at estrus and diestrus (P<0.01), with no difference between estrus and diestrus. The peptide level of IMD was higher at diestrus than those at proestrus and estrus (P<0.01).

### mRNA expression of *Crlr* and *Ramps* in cycling rats

**Figure 2:**
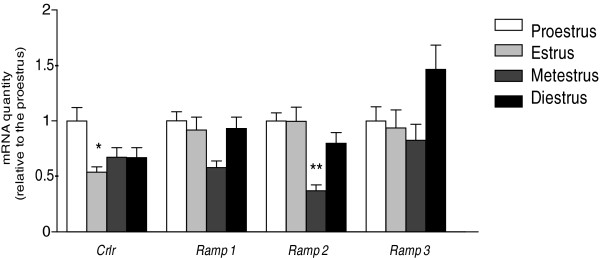
**Expression of uterine *Crlr*, *Ramp1*, *Ramp2*, and *Ramp3 *mRNAs across estrous cycle. **n=6, 6, 7, and 9 for the four stages respectively, * P<0.05 compared with the proestrus stage, ** P<0.05 compared with all the other stages.

The *Crlr*, *Ramp1*, *Ramp2*, and *Ramp3* mRNA levels estimated by real-time RT-PCR are shown in Figure 2. The *Crlr* mRNA level was significantly lower at estrus than that at proestrus (P<0.05) while the *Ramp2* level was lower at metestrus than all the other stages (P<0.05). No changes were observed for *Ramp1* and *Ramp3* mRNA levels.

### Gel filtration chromatographic analysis of the uterus

**Figure 3:**
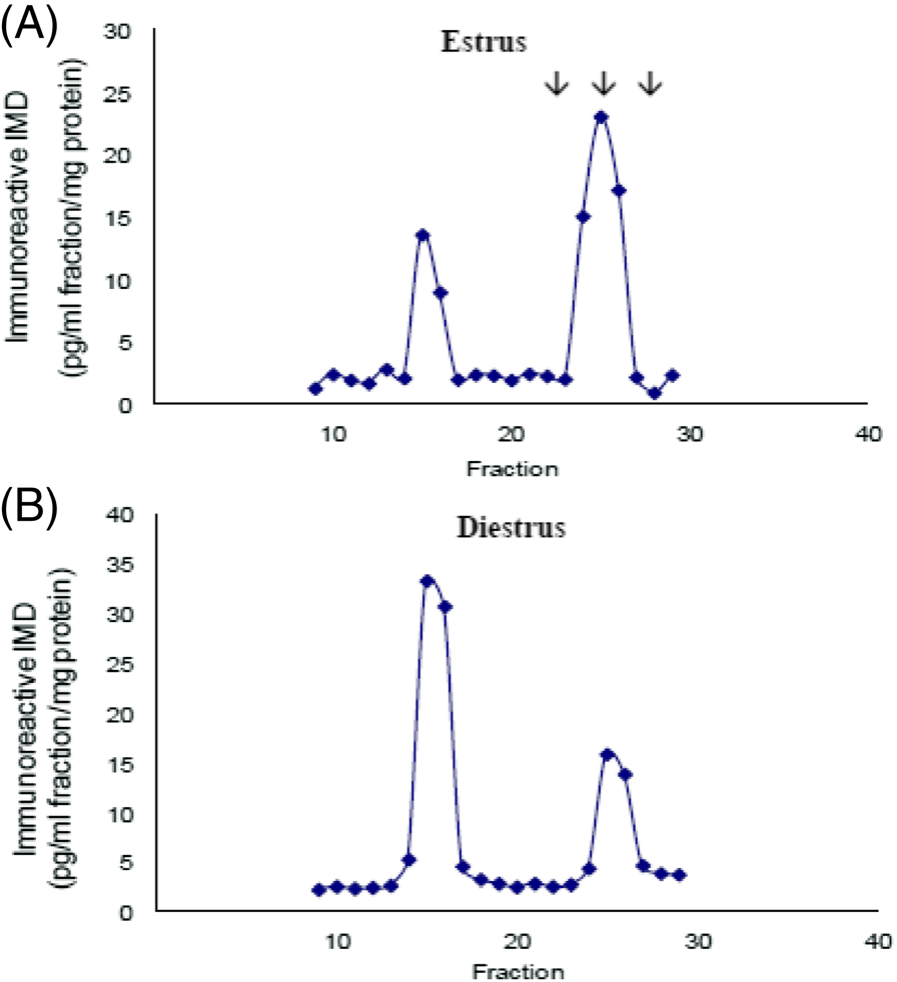
**Bio-gel P30 filtration chromatograms of the uterine extracts at estrus (A) and diestrus (B). **Mature IMD was eluted at fraction 25 and was similar in size to IMD_1-47_. The arrows indicate the positions where IMD_1-53_ (fraction 22)_,_ IMD_1-47_ (fraction 25) and IMD_8-47_ (fraction 27) were eluted. The peak ratios of IMD precursor: IMD_1-47_ are approximately 1:2 at estrus and 2:1 at diestrus.

At both the estrous (n=5) and diestrous stages (n=5), two immunoreactive IMD peaks were observed on the gel filtration chromatograms at fractions 15 and 25. At estrus, there was a higher peak of IMD than the IMD precursor while at diestrus, a predominant peak of IMD precursor and a smaller peak of IMD were seen (Figure 3). The IMD peak was much higher at estrus than at diestrus. Since the authentic IMD_1-53_, IMD_1-47_ and IMD_8-47_ were eluted at fractions 22, 25 and 27 respectively, the low molecular peak in the sample corresponded to IMD_1-47_.

### Immunohistochemical study of IMD

**Figure 4:**
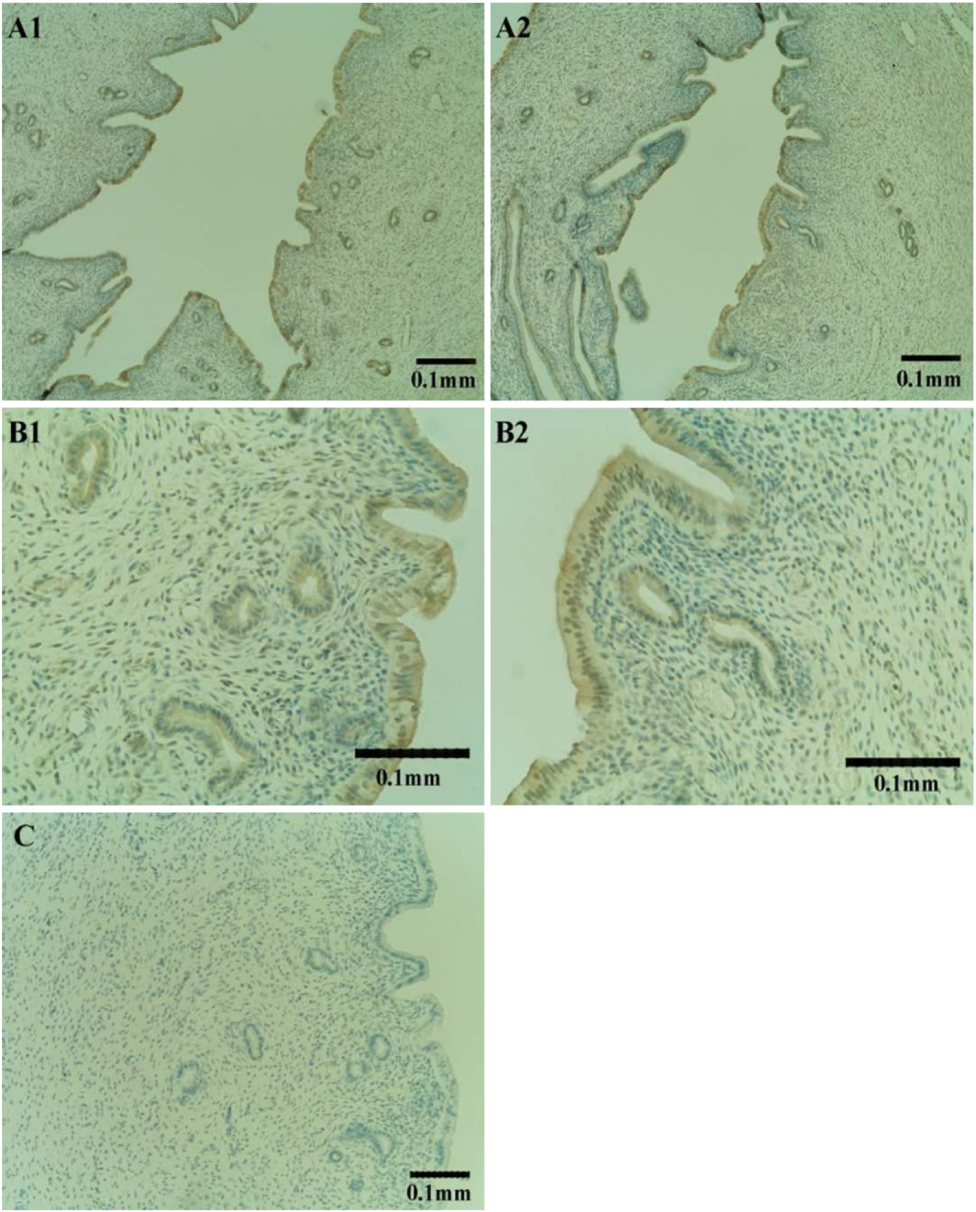
**Immunohistochemical study of IMD in the uterus. **Positive immunostaining at the luminal epithelium (**A**) and glandular epithelium (**B**) at both estrus (1) and diestrus (2); negative control without primary antibody (**C**).

Positive immunostaining was observed in the rat uterus at both estrus and diestrus in the luminal and glandular epithelial cells with similar intensities at the two stages (Figure 4).

### Effects of IMD and its receptor antagonists and inhibitors on uterine contraction

**Figure 5:**
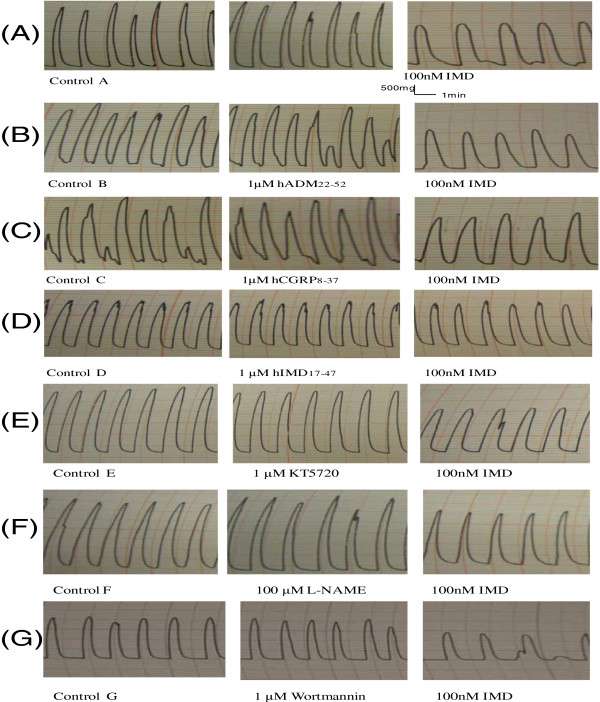
**Representative tracings of rat uterine muscular contraction *in vitro. ***Treatment of IMD alone (**A**), and together with receptor blockers hADM_22-52_ (**B**), hCGRP_8-37_ (**C**), or hIMD_17-47_ (**D**), or enzyme inhibitors of PKA (KT5720) (**E**), NO synthase (L-NAME) (**F**), or PI3K (Wortmannin)(**G**).

**Figure 6:**
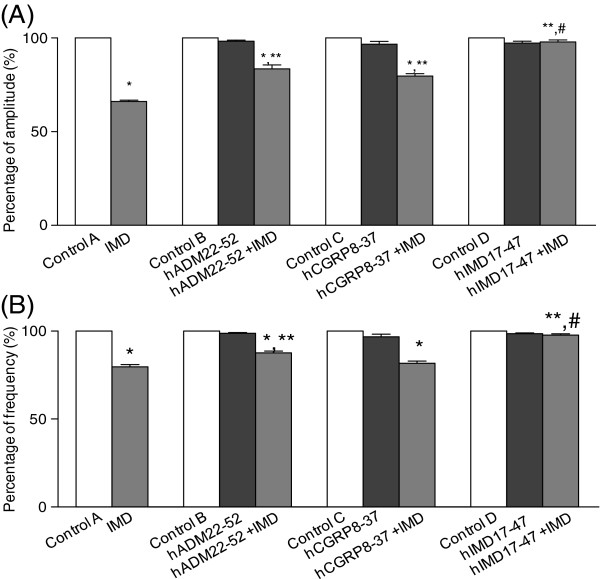
**The effects of IMD receptor antagonists on the inhibition of uterine contraction by IMD. **Decreased amplitude (**A**) and frequency (**B**) were observed after IMD treatment. Both hADM_22-52_ and hCGRP_8-37_ partially inhibited IMD actions. hIMD_17-47_ completely blocked the relaxation effect of IMD on both the contraction amplitude (**A**) and frequency (**B**). n=6 for all treatments. Data are presented as mean±SEM. *P<0.01 vs control or blocker alone, ** P<0.01 vs IMD, #P<0.01 vs hADM_22-52_+IMD or hCGRP_8-37_+IMD.

**Figure 7:**
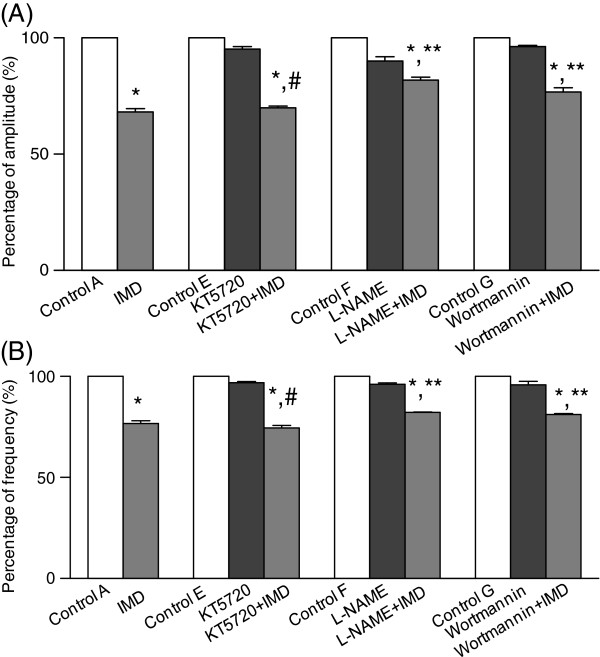
**The effects of inhibitors in signaling pathways on the suppression of uterine contraction by IMD. **Decreased amplitude (**A**) and frequency (**B**) were observed after the IMD treatment. Both L-NAME and Wortmannin partially blocked IMD actions (A and B). KT5720 had no effects on uterine contraction mediated by IMD. *P<0.01 vs control or inhibitor alone, **P<0.01 vs IMD, #P<0.01 vs L-NAME+ IMD or Wortmannin+IMD.

Representative tracings of the rat uteri treated with IMD, receptor antagonists and signaling pathways inhibitors are shown in Figure 5. IMD inhibited uterine contraction in both frequency and amplitude (Figure 5A). Treatment of rat uteri with IMD significantly lowered the amplitude and frequency of contraction by 33.67±0.78% and 20.34 ± 1.33% respectively (P<0.05, Figure 6A and B) but had no effects on the basal tone (results not shown). Both hADM_22-52_ and hCGRP_8-37_ partially blocked the action of IMD on the amplitude (Figure 5B and C) and the amplitude decreased only by 16.42 ± 2.01% and 20.27 ± 1.26% respectively (vs 33.67 ± 0.78% for IMD alone, P<0.05, Figure 6A). Only hADM_22-52_ but not the hCGRP_8-37_ partially blocked the IMD action on frequency and the decrease was only 12.42 ± 1.01% (vs 20.34 ± 1.33% for IMD alone, P<0.05, Figure 6B). hIMD_17-47_ completely blocked the relaxation effect of IMD on both amplitude and frequency (Figures 5D, 6A and B). The use of KT5720 did not alter the responses of the contraction amplitude and frequency to IMD (Figure 5E) while the effects of IMD were partially inhibited by both L-NAME and Wortmannin (Figure 5F and G). In the presence of L-NAME and Wortmannin IMD decreased the amplitude by 19.13 ± 1.27 and 23.28 ± 1.80% (vs 33.67 ± 0.78% for IMD alone, P<0.05, Figure 7A) and the frequency by 15.85 ± 2.2 and 16.92 ± 5.2% (vs 20.34 ± 1.33% for IMD alone, P<0.05, Figure 7B).

## Discussion

We have here demonstrated the changes of uterine IMD levels in the estrous cycle and the effect of IMD on rat uterine contraction for a better understanding of the possible roles of IMD in the female reproductive tract. IMD decreased uterine contraction, in line with the findings for ADM [25,26].Our study also confirmed that *Crlr*, *Ramp1*, *Ramp2*, and *Ramp3* are expressed in the uterus [26], suggesting that both ADM and CGRP receptors are present in this organ. Uterine IMD peptide level was higher at diestrus than at estrus with no change in gene expression. However, according to the gel filtration chromatogram, much of the IMD immunoreactivity at diestrus was due to the precursor and the amount of IMD_1-47_ was actually lower than at estrus. The relative abundance of the precursor IMD at diestrus may be explained by the decreased processing of the precursor protein or the increased secretion of the active IMD. As the *Imd* mRNA level at diestrus was not higher than at estrus, an increase in synthesis with a concomitant increase in release appears to be less likely. Rather, the smaller IMD peak at diestrus may be due to the decrease in its formation from the precursor. Our results suggest there is a difference in the proteolytic processing of the IMD precursor molecule. Regardless of the cause, an increase in the IMD_1-47_ at estrus would mean a greater IMD action compared with diestrus. This may be related to uterine contraction (the present study) or angiogenesis as intermedin is an angiogenic growth factor [40]. The significance of an increase of *Imd* mRNA at proestrus remains unclear.

We were able to demonstrate for the first time that IMD inhibited spontaneous uterine contraction in rat by reducing the contraction amplitude and frequency by 33.67 and 20.34% respectively. The magnitude of inhibition was similar to that reported for ADM on galanin-induced contraction [25], where the entire uterus was used, and was less than that reported for ADM on basal contraction [26], where uterine strips were used. This inhibitory effect of IMD was reversed by both ADM and CGRP receptor antagonists, as was previously reported for ADM [26]. However, there is one minor difference between our study and the study of Yanagita et al. [26] in the way to achieve synchronization of the physiological state in the female rat. We injected the immature female SD rats with PMSG while Yanagita et al. injected the mature female SD rats with estradiol, but both methods synchronized the rats to the estrous stage. This inhibitory effect was reported at estrus when the IMD_1-47_ level was higher and may synergize with the inhibitory effect of ADM.

We have not studied the IMD effects in induced uterine contraction. In galanin-induced contraction in the rat, ADM inhibited contraction via CGRP receptor only [25]. CGRP inhibited galanin-induced [25] and substance P-induced contraction in the rat [41], and KCl-induced contraction in the human [42] and these effects were mediated by the CGRP receptor. In some of the CGRP actions on the uterus, the NO pathway was involved [43], but not in others [44].

In this study, both hADM_22-52_ and hCGRP_8-37_ partially blocked the inhibitory effect of IMD on rat uterine contraction while hIMD_17-47_ exhibited complete inhibition. These results suggest that IMD modulate uterine contraction mostly by specific IMD receptor and partially by CGRP and ADM receptors. In addition, our study has shown that both NO and PI3K pathways are involved in IMD mediated uterine contraction. The use of L-NAME (NO blocker) and Wortmannin (PI3K blocker) significantly reduced the decreases in amplitude and frequency induced by IMD but KT5720 (PKA blocker) did not alter the IMD action. The L-NAME inhibition may be mediated by PKG, which is activated by cGMP in the NO pathway leading to the dephosphorylation of myosin light chain to relax the uterine smooth muscle [43]**.** Another NO-mediated IMD effect on contraction has been reported in the rat papillary muscle [45]. The PI3K pathway for inhibiting smooth muscle contraction has also been reported in gastrointestinal smooth muscles [46] and an involvement of PI3K pathway for an IMD effect can be found in endoplasmic reticulum stress [21]. The cAMP-PKA system is not involved as the use of PKA blocker had no effect on IMD actions although the elevation of cAMP production was one of the characteristic features in the early study of IMD [2].

## Conclusions

In conclusion, IMD and the gene expression of its receptor components are differentially expressed in the uterus across the estrous cycle. IMD inhibits uterine contraction by decreasing the amplitude and frequency. This inhibitory effect at estrus may synergize with the inhibitory effect of ADM when the IMD_1-47_ level was higher.

## Competing interests

The authors declare that they have no competing interests.

## Authors’ contributions

CWW worked on the collection of tissues, real time PCR, EIA, gel filtration chromatography, immunohistochemistry, contractility study by organ bath technique, performed the statistical analysis and drafted the manuscript. FT and WSO coordinated the project. FT and WSO are respectively the principal and co-investigators of the research and holders of the grant. Both FT and WSO assisted in the manuscript revision. All authors read and approved the final manuscript.
